# Prenatal diagnosis of de novo small supernumerary marker chromosome 4q (4q11-q12): A case report

**DOI:** 10.18502/ijrm.v19i5.9258

**Published:** 2021-06-23

**Authors:** Reza Mohammadi, Raheleh Taheri, Fatemeh Shahriyari, Farnaz Feiz, Zahra Mohammadi, Sadegh Shirian, Reza Raoofian, Abdorrasoul Malekpour, Reza Pazhoomand

**Affiliations:** ^1^Genetic Laboratory, Shiraz Fertility Center, Zargari St., Shiraz, Iran.; ^2^Shiraz University of Medical Sciences, Shiraz, Iran.; ^3^Pathobiology Laboratory of Ordibehesht Hospital, Shiraz, Iran.; ^4^Department of Pathology, School of Veterinary Medicine, Shahrekord University, Shahrekord, Iran.; ^5^Shiraz Molecular Pathology Research Center, Dr Daneshbod Pathol Lab, Shiraz, Iran.; ^6^Shefa Neurosciences Research Center, Tehran, Iran.; ^7^Legal Medicine Research Center, Legal Medicine Organization, Tehran, Iran.

**Keywords:** Prenatal diagnosis, Array CGH, Chromosome 4, Chromosome markers.

## Abstract

**Background:**

Small supernumerary marker chromosomes (sSMCs) are chromosomal fragments with abnormal structures found in patients with fertility problems and developmental delay. They may be detected in amniotic cell karyotypes. sSMCs are categorized as hereditary or de novo. Here, we describe a case of prenatal de novo 4q11q12 sSMC and its molecular cytogenetic features which had no apparent phenotypic abnormality.

**Case:**

The fetus of a 36-yr-old pregnant woman was detected positive for Down's syndrome (trisomy 21) at the 16${}^{\mathrm{th}}$ wk of gestation. Quantitative fluorescent polymerase chain reaction technique was applied for the rapid detection of numerical aneuploidy of chromosomes X, Y, 13, 18, and 21 microsatellites. Array comparative genomic hybridization (array CGH) technique was also conducted following the karyotype analysis of amniotic cells. The karyotype analysis was also done for the parents. Quantitative fluorescent polymerase chain reaction result revealed a male fetus with a normal chromosomal pattern, while the amniocentesis karyotype analysis identified a male fetus with a marker chromosome (47, XY, +mar), and the sSMC were existing in 100% of amniocyte metaphase spreads. The parents' normal karyotypes indicated that the sSMC was de novo. Array CGH analysis revealed a 6.48-Mb duplication at 4q11q12. Eventually, the parents decided to terminate the pregnancy by legal abortion.

**Conclusion:**

Our study highlights the importance of the application of array CGH in combination with karyotype analysis for rapid and precise prenatal diagnosis of partial aneuploidy region.

## 1. Introduction

Small supernumerary marker chromosomes (sSMCs) are chromosomal fragments with abnormal structures that may not be detected by banding analysis. sSMCs have a size similar to chromosome 20 or smaller and cannot be detected by routine banding pattern analysis (1, 2). While the frequency of prenatal sSMC with no evident origin has been reported as 0.075% and 0.044% in live births, it is seven times more (i.e., 0.288%) in mentally retarded cases and 0.125% in subfertile cases. About 23% of the cases encompass inherited sSMC which are commonly paternal (16% vs 7%). Worldwide, there are ∼2.7 × 10${}^{6}$ living sSMC carriers; 1.8 × 10${}^{6}$ have a de novo sSMC, while ∼70% of them are clinically normal (3–5). There are no definite sSMCs karyotype–phenotype correlations, and the phenotypes may range from normal to having dysmorphic features and/or developmental delay, depending on the involved chromosomal region, tissue distribution of the sSMC, and the level of mosaicism (2). Therefore, there is an urgent need for prenatal genetic diagnosis of new sSMCs to forecast the clinical consequences of sSMC and prevention of possible clinical outcomes (2, 6). In this study, we aim to report a rare case of prenatal diagnosed de novo sSMCs derived from the long arm of chromosome 4 [sSMC (4)] using array comparative genomic hybridization (array CGH) technique for the first time.

## 2. Case Presentation

**Figure 1:**
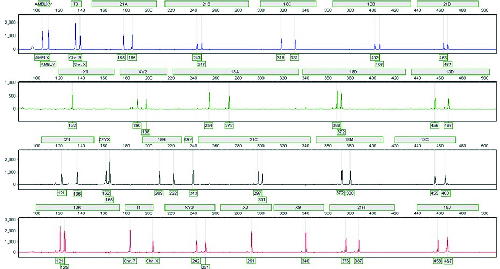
Quantitative Fluorescent polymerase chain reaction was performed for the fetus and the result is shown by electrophoretogram. Chromosomes X and Y, AMELXY, X1, XY2, ZFYX, XY3, X3, and X9 show a normal pattern of male karyotype (XY). T3 and T1 relative dosage comparison between chromosomes 3 and X with a peak ratio 2:1, indicating the presence of one chromosome X and two chromosomes 3. STR markers containing 21A, 21B, 21C, 21D, 21I, and 21H show two peaks (two chromosomes 21).

**Figure 2:**
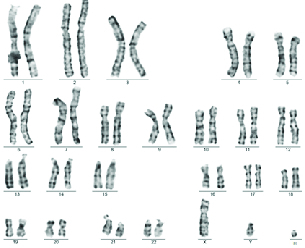
The amniocentesis G-banded karyotype showed the marker chromosome.

**Figure 3:**
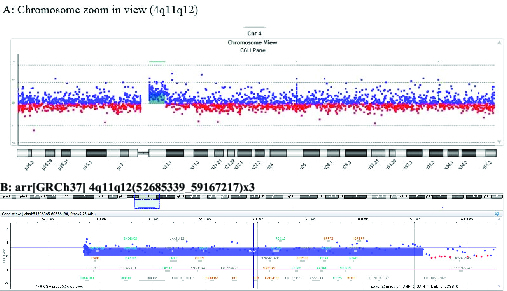
Array comparative genomic hybridization analysis of cultured amniocytes shows a 4q11q12 duplication. (A) Chromosome zoom-in view (chromosome 4) (1X enlarge) and (B) Genes located in region 52685339_59167217 of chromosome 4 (5X enlarge). (B) Chr4: 4q11q12 (52685339_59167217) x3 (duplication) [genome assembly GRCh37 (hg19)].

A 36-yr-old surrogate pregnant woman was detected to have a fetus with a high risk of Down's syndrome (trisomy 21) during the screening test at the 16${}^{\mathrm{th}}$ wk of gestation. All parameters of the ultrasonography scan at 11 wk and 3 days were normal, the nasal bone was present, and nuchal translucency thickness measured 1.4 mm. The results of biochemical analysis of maternal serum indicated that the risk of Down's syndrome was higher than that of the screening cut-off (1:30). The parents were phenotypically normal and there was no family history of congenital malformations. The amniocentesis was performed at the 16${}^{\mathrm{th}}$ wk of gestation. For the detection of numerical aneuploidy of X, Y, 13, 18, and 21 chromosomes, specific microsatellites were amplified using quantitative fluorescent polymerase chain reaction kit (Devyser CompactⓇ v3, Sweden). There was a male fetus, and the electropherogram did not reveal numerical aneuploidy in the mentioned chromosomes (Figure 1). GTG-banding analysis of 100 metaphase spreads showed same-sized sSMC in all primary amniocyte cultures and the fetal karyotype was detected 47,XY,+mar (Figure 2). Karyotype analysis was conducted on the peripheral blood of the biological parents (mother and father were 40- and 45-yr old, respectively) to determine the possible origin of the marker chromosome. There were normal karyotype patterns in all 30 examined cells. Array CGH technique was applied to identify the origin of the sSMC. Whole-genome array CGH was conducted on the DNA extracted from cultured amniocytes using Sure Print G3 ISCA V2 8x60K (Agilent Technologies, Santa Clara, CA, USA). The array comprises of 60,000 spots and identifies 500 established disease regions with the probe spacing median of ≥ 60 Kb. Array CGH analysis on the cultured amniocytes revealed a 6.48-Mb duplication at 4q11q12 (arr [GRCh37] 4q11q12 (52685339_59167217) x3) (Figure 3). The 4q11q12 duplication contains 33 OMIM genes, including 12 disease-causing regions such as SGCB, CHIC2, PDGFRA, KIT, KDR, SRD5A3, TMEM165, CEP135, SRP72, REST, SPINK2, and IGFBP7. Based on the mentioned findings, the parents decided to terminate the pregnancy at 18 wk and 5 days of gestation.

### Ethical considerations

The biological parents gave consent for amniocentesis and subsequent analysis and the use of the obtained results for publication.

## 3. Discussion

The impact of sSMC on prenatal genetic counseling has been a major challenge, is mostly based on theoretical data, and can be improved by the genotype–phenotype correlation studies and molecular cytogenetic analysis in which the chromosomal origins of the sSMC are detected (7). The chromosomal origin of sSMC must be detected to establish a reliable genotype–phenotype correlation (8). In our case, the prenatal molecular cytogenetic assay led to the detection of a de novo sSMCs derived from the proximal region of the long arm of chromosome 4 that resulted in trisomy of 4q11q12. The individuals carrying very small 4q11eq13proximal duplications seem healthy with normal features, however, they may have learning disability and developmental delay (4). However, the duplications of the proximal region of 4q have been contributed to different abnormalities and clinically important features. For example, a 4q12eq13 duplication in a 6-yr-old girl with microcephaly, facial dimorphism clinodactyly of the fifth finger, and psychomotor retardation has been detected (9). A 47,XY,+r [4] (::p10/q12::) karyotype in a 27-yr-old male with facial dimorphism, severe mental retardation, language disability, syndactyly of foot, as well as clinodactyly of the hand has been reported (10). A 15-yr-old girl with 4q13.1eq22.2 duplication, who had minor physical anomalies and moderate intellectual disabilities has been reported (11). A 2-yr-and-8-month-old boy with duplication of 4q12eq13 who had microcephaly, mild facial dimorphism, and mental retardation has been previously reported (12). Bonnet and coworkers reported a 6-yr-old obese girl with a developmental delay who had 82% mosaicism for an sSMC [4] 4q10eq13 in peripheral lymphocytes (13). An 8-yr-old girl with 8.6-Mb duplication of 4q13.1eq13.3 with developmental delay, attention-deficit hyperactivity, and speaking disability has been previously reported (14). A 47, XX, +mar has been detected with 4p11eq12 sSMC in which only long philtrum and hypertelorism were observed at the termination of pregnancy (15). A 3-yr-old boy with 4p11eq12-derived sSMC [4] presenting developmental delay, mild motor retardation, and mild hypotonic features has also been shown (7). As mentioned, de novo sSMCs were not indicated in any of the aforementioned studies, and such de novo sSMCs may be undetected causing major clinical manifestations. Our study provides useful information for genetic counseling on prenatally detectable sSMC of 4q11q12.

## 4. Conclusion

It has been concluded that if the marker chromosome is seen in the amniotic fluid sample but does not appear in parents, the CGH array is needed for making the best decision. Such findings help us in concise genetic counseling and guidance of couples making proper decisions about their fetuses.

##  Conflict of Interest

The authors have no conflicts of interest relevant to this article.
